# Homeownership While Aging—How Health and Economic Factors Incentivize or Disincentivize Relocation: Protocol for a Mixed Methods Project

**DOI:** 10.2196/47568

**Published:** 2023-07-10

**Authors:** Steven M Schmidt, Susanne Iwarsson, Åsa Hansson, David Dahlgren, Maya Kylén

**Affiliations:** 1 Department of Health Sciences Lund University Lund Sweden; 2 Department of Economics Lund University Lund Sweden

**Keywords:** housing market, aging-in-place, housing policy, healthy aging, mobility, housing preferences

## Abstract

**Background:**

Many factors influence housing choices among older adults, but far from all have been identified. There is little systematic analysis that has included economic factors and virtually no knowledge about the interplay among perceived costs of moving, health status, and the mobility rate of older homeowners. It is currently unclear whether economic factors influence older adults’ willingness to move, and the effects of economic policies on their actual behavior in the housing market are largely unknown.

**Objective:**

The overarching objective of the AGE-HERE project is to develop knowledge of the relationship between health and economic factors that incentivize or disincentivize relocation during the process of aging.

**Methods:**

This project uses a mixed methods convergent design across 4 studies. The initial quantitative register study and subsequent qualitative focus group study will nurture the evidence base and the development of a national survey. The final study will synthesize and integrate the results of the entire project.

**Results:**

Ethical approval for the register study (DNR 2022-04626-01) and focus group study (DNR 2023-01887-01) has been obtained. As of July 2023, data analyses (register study) and data collection (focus group study) are currently being conducted. The first paper based on the register data is expected to be submitted after the summer of 2023. Three meetings have been held with the nonacademic reference group. The qualitative data will be analyzed in the autumn. Based on the results of these studies, a survey questionnaire will be developed and distributed nationally during the spring of 2024, followed by data analyses in the autumn. Finally, the results from all studies will be synthesized in 2025.

**Conclusions:**

Results from AGE-HERE will add to the knowledge base for research on aging, health, and housing and can play a critical role in guiding future policy decisions aiming to balance the housing market. Such developments may lower related social costs and support older adults to maintain active, independent, and healthy lives.

**International Registered Report Identifier (IRRID):**

DERR1-10.2196/47568

## Introduction

### Background

The aging population increases the demand for housing adapted to diverse needs that facilitates active and healthy aging [1]. However, this demand is far from met because older adults face a challenging housing market with few choices available [2]. In Sweden, the supply of newly built homes has not kept pace with the increasing demand, and municipalities experience housing shortages [2], which has a major impact on individuals as well as on economic growth. Reforms have been implemented, and further reforms have been discussed. However, the housing policy debate has largely been based on increasing housing construction rather than finding ways to make better use of the existing housing stock.

According to the public debate, older homeowners are concerned about the costs associated with moving (eg, higher rental costs, a monthly association fee, and bank policies) [3]. At the same time, the housing market is experiencing insufficient residential mobility and inefficient relocation chains [2]. However, studies on housing and health in later life seldom incorporate economic factors [4], and how such factors influence older adults’ willingness to move is currently unknown. A better understanding of these relationships can play a critical role in guiding future policy decisions aiming to balance the Swedish housing market, which in turn may lower associated economic and social costs as well as support older adults to maintain active, independent, and healthy lives.

Older adults spend more time at home compared to other age groups, and therefore, it is important that the home environment is adapted to suit their needs and preferences. Thus, as people age, housing is associated with health outcomes [5-8]. For example, accessibility problems in the home may lead to injuries and social isolation, which in turn have negative health effects [9]. Yet, research on factors that contribute to residential mobility for those who wish or need to move is sparse [8]. Given that older adults represent a group with varying housing preferences and needs [10,11], more knowledge is needed to support the development of future housing policies that accommodate this heterogeneity.

Recent international research has primarily focused on relocation to residential care facilities [12-14], with disabilities and frailty as predictors of relocation [15]. Situational circumstances such as strong community ties and individual or family health, in combination with social and economic conditions, make housing decisions complex in older age [16]. Thus, negotiating relocation is described as a long process until turning points emerge [17,18]. While these studies primarily focus on the very old, a recent study using register data from the United States showed that factors such as a change in income, years living in the community, and degree of social support are important characteristics for predicting relocations among persons in early retirement age [10]. Adding to this, qualitative research from Sweden suggests that older adults start to have more active reflections regarding their future housing choices and relocations around retirement age [19]. While some studies indicate that retirement triggers actual moves [10,20], we do not know whether economic issues encourage this younger group of older adults to stay in privately owned houses longer, even though those no longer suit their needs and preferences. This knowledge is crucial, especially during times of economic uncertainty and rapid social change, as it can assist policymakers in making informed decisions that support older adults in making suitable housing choices and enhancing their overall well-being.

Turning to the economic situation on a national level, macroeconomic trends such as booms or recessions, usually measured by gross domestic product growth, are likely to influence individuals’ behavior in the housing market. This might be especially true for homeowners, as they often have most of their savings invested in the house and can therefore be affected by changes related to the housing market, such as house prices and interest rates [21]. Yet, the majority of studies on this topic focus on the entire population [22] rather than specific age strata, such as older adults. One exception is a study conducted in the United States evaluating lock-in effects due to the financial crash in 2008 and a subsequent major decrease in housing prices. The authors found that the probability of downsizing for adults aged 55 years and older was higher among people with a higher loan-to-value ratio (measured by mortgage loans or value of the house) compared with owners with a lower ratio [23]. Hence, it is likely that economic fluctuations influence households differently, but how such associations develop over time and emerge in a Swedish context remains unknown.

### Housing Policy Context

The AGE-HERE project focuses on homeowners aged 55 years and older. In Sweden and most other Organisation for Economic Co-operation and Development (OECD) countries, the most common form of tenure among this age group is single-family home ownership [24,25]. This age group often has most of their savings invested in their house, and other available assets are limited [21], which makes them more vulnerable to changes related to the housing market, such as interest rates or housing prices. As to other economic factors, several have been suggested to encourage this population segment to stay in their current home rather than move. The 2008 Swedish property tax reform lowered the yearly property tax on especially highly assessed property by introducing a low maximum amount, but the capital gains tax was raised from 20% to 22%. Taken together, these changes increased the incentives to stay in large houses rather than moving to smaller ones. In addition, rent control has reduced the supply of apartments, which increases the price of other types of housing, such as owner-occupied housing [3]. Hence, it is likely that the way housing is taxed influences people’s relocation behavior, which calls for more research in this area.

### Study Objective and Research Questions

AGE-HERE aims to develop new knowledge on relationships between economic factors and health that incentivize or disincentivize relocation among homeowners from an early stage of the aging process (55 years and older). The specific research questions are as follows:

- How did macroeconomic events during the period 1970-2016 affect relocation patterns across time for older homeowners of different ages? Are there different patterns of relocation depending on civil status and sex?
- In what way did the tax reform of 2008 affect the probability of moving among older homeowners?
- What economic factors predict relocation to different types of housing (eg, condominiums, owner-occupied housing, and rental apartments), taking health factors into account?
- What economic and health factors do older homeowners perceive as important when considering relocation?
- What are older homeowners’ preferences and beliefs regarding the economic factors that disincentivize or incentivize relocation?
- To what extent do older homeowners report that economic factors are an important part of the relocation decision process?
- How are economic factors and health related to decisions to move among older homeowners?
- How are the questions above affected by regional differences and housing market structures?

### Framework

As a starting point, we will use a framework developed by Roy and coauthors [4] to conceptualize and examine the complexity of factors associated with housing decisions in older age. Covering 6 dimensions—socioeconomic and health, built and natural environment, social, time and space, psychological and psychosocial, and housing economics—this framework is a useful tool to build ties between different research fields [26]. With this framework as the starting point, we will combine several bodies of theories used in research investigating housing decisions in older age: environmental press, migration theory, residential normalcy, and control [4].

Deciding to relocate or not is influenced by individual health factors such as functional limitations and environmental factors [4], which is in line with the environmental press theory stating that the relationship between a person and their environment (social, environment, policy) affects their well-being and behavior [27,28]. Additionally, migration theory covers several domains described by Roy et al [4] and is important to elucidate the place- and people-based push and pull factors that influence older adults’ decisions to relocate, particularly after retirement [29]. For example, while investment and housing value may be important for the decision to remain in a current home, stressors such as poor health, safety concerns, mortgages, and tenure status might manifest as push factors leading to a decision to relocate. In parallel, we will draw upon theories relating to the concepts of space (ie, housing, the physical dwelling) and place (ie, a home, with personal meanings) [28]. Thus, the meaning assigned to a home impacts the decision to relocate, and moving into a new home initiates a process of recreating meaning in the new place through living there [28]. These are important aspects in the time and space dimensions as well as in the psychosocial dimension of Roy et al’s [4] framework. From an economic perspective, we will use the life cycle hypothesis, which predicts that households favor smooth rather than uneven consumption over their life cycle. In line with the economic factors (eg, mortgages, relocation costs, and taxes) described as important in the framework, the life cycle hypothesis implies that older adults who view their property as part of their retirement savings would sell their property in favor of renting or moving to a smaller, less costly home (ie, to increase the possibility to consume). In addition, tax theory, specifically the principles of tax neutrality and economic efficiency [30], is relevant. For taxes to be efficient, they should be designed so they do not influence behavior (eg, consumption and investments) [30], but when diverse types of housing are taxed differently (as in Sweden), taxes will steer behavior toward types of housing with beneficial taxation. That is, in contrast to the prediction of the life cycle hypothesis described above, the Swedish tax system may disincentivize relocation, so older adults remain in their homes even if that is not what they prefer.

## Methods

### Overview

**Figure 1:**
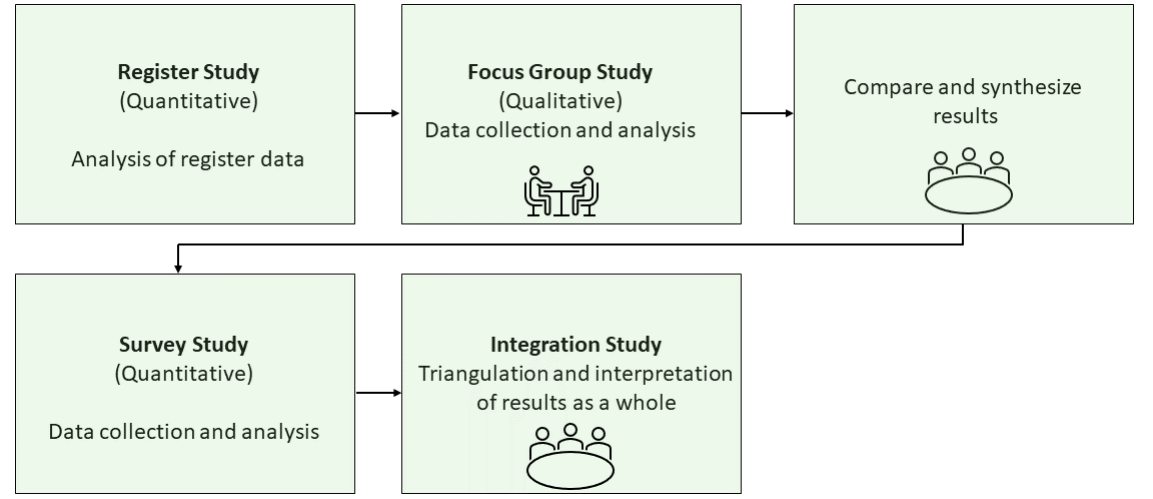
AGE-HERE project design; mixed methods convergent design.

AGE-HERE has 4 parts (Figure 1): a register study, a qualitative focus group study, a national survey study, and an integration study. The project is conducted in collaboration with a reference group consisting of nonacademic partner representatives (eg, housing market experts and senior citizen organization representatives) who will provide their input and reflections throughout the research process.

### Study Design

AGE-HERE has a mixed methods approach in a convergent design [31]. Results from the initial quantitative and qualitative studies will be used to develop a quantitative national survey. The final study will synthesize and integrate the results of the entire project (Figure 1).

### Register Study

To investigate how macroeconomic events and taxes on property and capital gains affect relocation patterns for older homeowners, we will use data from several registers such as the Longitudinal Individual Data Base, a longitudinal database with a representative sample of the population in Sweden (N=300,000) from 1968 to 2016, and the income and taxation register, which provides information related to taxation and income for the entire population of Sweden. To analyze different factors, we will link data from the Longitudinal Individual Data Base and the income and taxation register to register data on health and housing, for example, from the property register, the population register, and the patient register.

### Analysis

#### Tax Reform 2008

Differences-in-difference analysis [32] will be used to study the effects of the tax reform of 2008. Detailed information about taxes and income sources, as well as types of housing and socioeconomic variables, will be used to compare households that were affected to a large extent by the tax reform, that is, those living in highly assessed properties (high exposure group), with those that were less affected, that is, those living in properties worth less (low exposure group). The differences in the number of moves between the high and low exposure groups 2 years prior to the reform (2006) with the differences in the number of moves in the same groups 2 years after the reform (2010) will be compared. As this is not a randomized experiment, 3 methods to adjust for potential systematic differences between the groups will be used. First, we will control for variables such as income, education level, and place of residence that may affect the 2 groups differently. Second, we will use a propensity score matching technique [33] to determine a matched comparison group. Third, we will use a regression discontinuity design to evaluate the effect of the 2008 tax reform. Because the 55 years and above age group is heterogeneous, we will investigate whether different groups are more or less sensitive to economic factors. More specifically, we will compare different subgroups such as age cohorts, retired or working, have been or are receiving sickness payments, marital status, and sex.

#### Macroeconomic Trends and Health Factors

To evaluate historical trends in the relocation patterns of older adults, descriptive statistics will be used. Means will be described over time for different groups of individuals, depending on age and macroeconomic characteristics for the specific year of the relocations.

To analyze how health factors influence relocation, we will use descriptive and inferential statistics such as odds ratios, mean comparisons, and multivariate regression analyses.

### Focus Group Study

To explore how older homeowners reason about their current housing situation in relation to future needs and opportunities to move and what financial and health-related factors they perceive as important in such a decision-making process, data will be collected from a series of focus groups [34] in 4 municipalities across Sweden.

#### Selection of Municipalities and Recruitment of Participants

Four heterogeneous municipalities have been selected based on demographics (ie, proportion of older adults), local housing market structure (eg, range of housing types, price, rent levels, and new production of housing that matches the needs of older adults), and geographic location.

Participants will be recruited via senior citizens’ organizations [19] and through advertisements on social media. The selection of interested participants will be based on similarities dictated by the purpose of the study [34] and heterogeneity among them (eg, sex, age, and living situation) to explore various perspectives. A total of 64 people will be recruited into 8 groups (ie, 2 focus groups per municipality), with 8 participants in each.

#### Data Collection

The interview guide will be developed based on findings from the register study, theories underpinning the project, relevant literature, and current media discourse. The groups will meet twice each, and the preliminary results from the first sessions will be used to adapt the interview guide for the second session to allow deeper exploration of the themes that emerge. If a need to contrast different perspectives emerges in the analysis, the participant constellations may change during the second session. Two moderators will lead each group: one will lead the discussions, and one will observe group interactions and listen to the discussions [34]. Sessions will be audio-recorded and last approximately 2 hours. The observing moderator will take field notes on settings and group dynamics as well as pose clarifying questions. At the end of each session, the observing moderator will give a brief summary of the core issues discussed and allow participants to comment and suggest amendments.

#### Analyses

We will use a constant comparative analytic framework as described by Krueger and Casey [34]. After round 2, audiotapes will be transcribed verbatim. The moderators will read the transcripts and listen to the recordings to get a feel for the dynamics of the discussions and independently code the data. This includes identifying patterns in the data and relationships among concepts. NVivo (Lumivero) will be used as it promotes transparency and validation of coding. To further enhance trustworthiness and reduce potential bias, peer debriefing meetings with all coauthors will be held throughout the analysis process [35].

### Survey Study

#### Overview

The survey study will include a random sample of homeowners aged 55 years and older living across Sweden. In Sweden, approximately 3.71 million people are aged 50 years or older. Within this group, 2.8 million (70%) live in privately owned accommodations: 2.13 million in houses and 710,000 in condominiums [36]. Because men own their homes to a greater extent than women of older age, participants will be stratified by sex and randomly selected from the Swedish national population registry. Using a 95% CI and a margin of error of 4, we estimate a total sample size [37] of 3600 to be representative. We expect a 40%-50% response rate and will therefore draw an initial sample of 7200.

#### Survey Data Collection Procedures

Results from the register study and focus group study will be used to develop the survey questions in an iterative process involving the nonacademic reference group. The reference group will provide input to ensure the survey length is reasonable, the questions and response choices are clear and understandable, and the items cover the content identified in the previous studies. Sociodemographic questions and established self-rating scales on quality of life, independence, participation, and health will be integrated into the survey to explore the relationships between personal factors, health, and economic factors in decisions to relocate. The survey will be tested in a pilot with a sample drawn from the target population for the main study (n=25), and questions and procedures will be modified based on the results.

A professional survey company will be contracted for the data collection [19]. Potential participants will be sent a postal invitation, which includes a detailed description of the study and instructions for how to opt out. Participants will be able to complete the survey via the internet, using a paper form, or via telephone. Those who have not completed a web-based survey or requested a paper survey after 2 weeks will be contacted by trained interviewers who can conduct the survey using a computer-assisted telephone interview protocol. Data collection will continue until the target sample size is reached.

#### Analyses

Descriptive statistics will be used to analyze the demographics of each stratum and compare them with national population statistics. If results differ from the population, sample weights to match the population will be created. Weighted sample statistics will be used to describe the prevalence of reported economic incentives or disincentives, and a 2-tailed *t* test will be used to identify sex differences. Univariate and multivariate linear and logistic regression models will be used to assess associations between economic factors, health, and sociodemographic variables.

### Integration Study

As a final step, toward the end of the project (in 2025), the results will be merged and interpreted as a whole [31]. The purposes are triangulation (ie, validation based on corroboration between data sets), complementarity (ie, different methods to answer related questions), and expansion (ie, data sources address different aspects of answering questions raised by other data sets) [38,39]. Using an interactive merging strategy, narrative integration and weaving, presenting qualitative and quantitative data together, and forming connections based on specific topics will be used [40]. Analysis meetings involving the reference group will be an integral part. Emerging themes will be explored and developed based on Roy et al’s [4] framework, the theoretical underpinnings, relevant international or national reports, policy documents, and legislation.

### Ethics Approval

Ethical approval for the register study (DNR 2022-04626-01) and focus group study (DNR 2023-01887-01) has been obtained from the Swedish Ethical Review Authority. We will adhere strictly to the Helsinki Declaration’s requirements for research involving humans. Because a systematic examination of ethical considerations is also needed in the survey study, an additional ethical approval will be sought. We will make sure that project staff are trained and supervised on ethical issues, with specific attention to collecting data from older adults. In case participants have questions, contact information of the responsible researchers will be provided for the focus group study and the survey study. In the information letter to study participants, they will be informed about confidentiality and their right to withdraw from the study at any time. Informed consent will be obtained first after the participants have been given written and oral information on the study’s purpose and what participation would entail. All data will be collected and stored in compliance with the requirements of the Ethical Review Authority and the European General Data Protection Regulation.

## Results

Ethical approval for the register study and focus group study has been obtained (DNR 2022-04626-01, DNR 2023-01887-01 respectively). Data analyses (register study) and data collection (focus group study) are currently being conducted (July 2023). The first paper based on the register data is expected to be submitted during the summer of 2023. Three meetings have been held with the nonacademic reference group. The qualitative data will be analyzed in the autumn. Based on the results of these studies, a survey questionnaire will be developed and distributed nationally during the spring of 2024, followed by data analyses in the autumn. Finally, the results from all studies will be synthesized in 2025.

## Discussion

### Principal Findings

This project sets out to investigate how economic and health aspects influence the decision to relocate or not among older adults. By integrating theory and methods from economics and gerontology, we will identify the most influential factors in older homeowners’ housing decisions. We expect that economic policies play an important role in the decision to remain in single-family homes even when the home no longer supports older adults with declining health. Furthermore, we expect this decision to also be related to a lack of affordable housing with good accessibility in the Swedish housing market. Although we specifically focus on Sweden, the project has broader geographical implications. Hence, findings can be used to inform the allocation of resources and policy decisions concerning the living arrangements and housing choices of older adults in countries where comparable housing policies are being implemented or intended.

Aging in place has become the norm for most older adults in Sweden as well as in other Western countries, which is supported by society as it is considered less costly to provide home care than care in institutions [41]. Even so, most research on housing and aging does not consider factors that incentivize or disincentivize relocation within the ordinary housing stock; instead, the focus is on people aging with disabilities or frailty, with growing needs for special forms of housing and housing adaptations [14]. Given that aging in place is far from supportive for all older adults [42], future research and policy initiatives should focus on the diverse needs of the heterogeneous aging population [43] and consider aging in the right place [44]. This is important because, with population aging, the share of older adults living with functional limitations and age-related diseases will increase [45], implying that the housing stock needs to be adapted and developed to fit the needs and preferences of this substantial population segment. The dwellings older homeowners tend to live in often have a high prevalence of environmental barriers, and older adults aging with functional limitations experience accessibility problems, which can hinder daily activities and have negative consequences for health and quality of life [9,46,47]. From a societal economic and public health perspective, it is therefore important that municipalities comply with their housing provision responsibility and create conditions for everyone to be able to live in adequate housing [48].

Most older adults in Sweden live in dwellings they purchased much earlier in their lives and are now reticent about selling [49]. This trend is similar to the majority of OECD countries [25]. For example, in the United States, homeownership rates remain unchanged after the age of 55 years [50], and this trend has been relatively stable despite the economic recession and the housing market crash in 2007 [44]. The reluctance to sell contradicts the life cycle hypothesis, which says that older homeowners would sell their property in favor of renting or moving to a smaller, less costly home to start “consuming” or using their equity to support their living costs [50]. While many factors can explain why older homeowners are reluctant to move (eg, attachment to a place), concerns about the associated costs have gained little attention. This has been highlighted by Sweden’s largest senior organization, showing that every fifth senior household in Sweden has decided not to move because of the capital gains tax [49]. This means that a substantial number of moves do not happen, which in turn slows down relocation chains and reduces the housing supply for young families who are in need of a larger home.

While there are other recent reports highlighting this issue [51], no scientific efforts have so far been made in Sweden or internationally to understand this complexity in depth. Overall, there is a paucity of studies investigating associations between taxation and decisions to move. Previous studies are based on US conditions and aggregated data [52], where the property tax is local and used to finance local public services and therefore affects the choice to move. As such, the AGE-HERE register study represents a novel approach compared to existing research, as we will study the impact of taxes on housing decisions in older age using high-quality register data. The design of the Swedish property tax, which is national rather than local (and therefore does not interfere with the choice to move), allows us to test this empirically in a valid way. By linking registers with data on taxes, income, and health and using a framework based on a systematic literature review [28], we will be able to analyze a large range of factors. Adding to this, we will observe the number of relocations among older adults historically, which will provide unique knowledge on housing decisions depending on the state of the economy across time.

Turning to the focus group study, previous research conducted in 6 European countries (the United Kingdom, Germany, the Netherlands, Ireland, Italy, and Hungary) has shown that housing choices among older homeowners are highly dependent on the local housing market situation [53], which is seldom considered in research. Our approach in AGE-HERE to collect data in 4 different municipalities across Sweden will provide valuable insights related not only to the range of possible factors that influence older homeowners’ decisions to move but also to the role played by preconditions in local housing markets. Local housing markets are heterogeneous and need to be considered by housing policymakers. For example, in some municipalities, almost the entire housing stock consists of single-family houses, which limits the possibility of moving to a smaller dwelling when needs and preconditions change. In contrast, other municipalities have a large variety of smaller dwellings, both rentals and condominiums. In terms of new production, especially forms of housing that are aimed at the older population, some municipalities have many completed and ongoing projects while others have few. It is not farfetched to believe that such diverse situations will be a huge challenge in the coming years as the share of older people will increase substantially in all municipalities. That is, housing needs will change with population aging, and therefore policymakers need to relate to this diversity of prerequisites when planning for the future.

By better understanding the living conditions of older people from their own diverse perspectives, we can promote a positive development of social relations and enhance full participation in society on an individual level. Additionally, a better functioning housing market would have positive spillover effects on the labor market because relocation chains would function better and on the financial markets because real estate prices would be adjusted, which in turn would affect households’ debt levels [3]. Altogether, a better-functioning housing market will enhance not only health and well-being but also the economic performance of society.

### Limitations and Strengths

AGE-HERE is conducted in Sweden, but the knowledge gained from the project is expected to be relevant for the development of housing and policies internationally. Hence, population aging is a global phenomenon, and most countries will need to adapt their housing markets to fit diverse needs and expectations.

The data collection for the AGE-HERE focus group and survey studies will be conducted against the backdrop of the COVID-19 pandemic, during a time of high energy prices and inflation. How such periodic effects may influence the data collected will be considered and discussed in all parts of the project.

### Conclusions

Results from AGE-HERE can inspire future research and play a critical role in guiding policy decisions aiming to balance the housing market, which in turn may lower related social costs and support older adults to maintain active, independent, and healthy lives.

## Appendix

Multimedia Appendix 1 Peer review report from Länsförsäkringar.

## Abbreviations

OECD: Organisation for Economic Co-operation and Development

## Data Availability

The data sets generated and analyzed during the register and survey studies are not publicly available due to a data agreement between Lund University and a third party but are available from the corresponding author or data holder on reasonable request.
