# Supplementary material for: Homeownership While Aging—How Health and Economic Factors Incentivize or Disincentivize Relocation: Protocol for a Mixed Methods Project
Source: JMIR Res Protoc. 2023 Jul 10;12:e47568. doi: 10.2196/47568 (PMC10366958; doi:10.2196/47568)
Supplement: Multimedia Appendix 1 [file resprot_v12i1e47568_app1.pdf]

Evaluation and Assessment: Research financing grant - project designation number P7/21

Project title:
AGE-HERE: Homeownership while ageing - how do health and economic factors incentivize or disincentivize relocation

Project duration:
2021-01-01 - 2025-12-31

Sought grant:
3 997 405 SEK

Awarded grant:
6 019 934 SEK

Principal investigator (PI):
Maya Kylén

Institution:
Department for Health Science, Faculty of Medicine, Lund University

Evaluation and reasoning

Evaluating criteria:

Originality (scale 1–5):
5 – Exceptional/Outstanding: exceptionally orginal application

Scientific quality (scale 1–5):
5 – Exceptional/Outstanding: exceptionally strong application

PI's and co-applicants' competence (scale: 1–5):
4 - Excellent

Viability/Operationality (scale: 1–3)
3 – Viable/Operational: the application raises only negligible concerns regarding the project's viability/operationality.

Relevance to the funding call (scale: 1–3)
3 – Relevant: the application's aims and objectives are highly relevant to the funding call's interest areas and priorities.

Relevance to society, users, and stakeholders (scale: 1–3)
3 - Relevant: the application takes into consideration the knowledge needs of users and stakeholders outside of academia and has comprehensive plans for disseminating results and for impact outside of academia.

Relevance for Länsförsäkringar (scale: 1–3)
3 – Relevant: the application is of high relevance to Länsförsäkringar's knowledge needs and the expected results are deemed transferable.

Länsförsäkringar

Application's overall evaluation:
6 – Exceptional/Outstanding

Reasoning:
The application aims to investigate how economic, behavioral and health factors effect older homeowners housing choices and in turn their mobility in the housing market. The application is judged as exceptionally strong, original, and well-motivated, as well as highly likely to advance science and contribute more generally. The application's research design and methods are judged as well thought-out and relevant for answering the stated research questions. The research group is deemed to have the required competence both in relation to researching the stated research questions and in using a multi-disciplinary methodological approach. The application is judged of additional importance given the stated desire to develop (policy) recommendations. During the evaluating committee meeting the principal investigator was recommended to consider including: 1) a larger geographical spread in the study design, and 2) a greater number of economic/financial policy reforms.

The principal investigator has taken these recommendations into consideration and resubmitted the application. With regards to the first recommendation, the research group will now collect and analyze both quantitative and qualitative data from a larger geographic area. With regards to the second recommendation, the research group will now investigate a greater number of economic/financial policy reforms, including how the RUT and ROT reforms have incentivized and/or disincentivized older homeowners' relocation choices. The adjustments made to the application meet the recommendations suggested by the evaluating committee. As a result of these adjustments, the application is judged to have further increased its scientific quality as well as its scientific contribution and more general relevance. Fulfilling the recommendations has budgetary implications, with a budget increase from the sought research award of circa four million Swedish Kronor to just over six million Swedish Kronor. Länsförsäkringar's Research Foundation's board approves the resubmitted application and budget and awards a research grant of 6 019 934 Swedish Kronor.
